# Role of Sea Surface Physical Processes in Mixed‐Layer Temperature Changes During Summer Marine Heat Waves in the Chile‐Peru Current System

**DOI:** 10.1029/2021JC018338

**Published:** 2022-07-15

**Authors:** Kylene M. Cooley, Melanie R. Fewings, James A. Lerczak, Larry W. O’Neill, Kevin S. Brown

**Affiliations:** ^1^ College of Earth, Ocean, and Atmospheric Sciences Oregon State University Corvallis OR USA; ^2^ Department of Chemical, Biological, and Environmental Engineering Oregon State University Corvallis OR USA; ^3^ Department of Pharmaceutical Sciences Oregon State University Corvallis OR USA

**Keywords:** heat budget, Chile Peru Current System, warm anomaly, winds, sea surface temperature, mixed‐layer

## Abstract

We identified anomalously warm sea surface temperature (SST) events during 1980–2019 near the major upwelling center at Punta Lavapié in the central Chile‐Peru Current System, using the European Centre for Medium‐Range Weather Forecasts reanalysis and focusing on time scales of 10 days to 6 months. Extreme warm SST anomalies on these time scales mostly occurred in the austral summer, December through February, and had spatial scales of 1000s of km. By compositing over the 37 most extreme warm events, we estimated terms in a heat budget for the ocean surface mixed layer at the times of strongest warming preceding the events. The net surface heat flux anomaly is too small to explain the anomalous warming, even when allowing for uncertainty in mixed‐layer depth. The composite mean anomaly of wind stress, from satellite ocean vector wind swath data, during the 37 anomalous warming periods has a spatial pattern similar to the resulting warm SST anomalies, analogous to previous studies in the California Current System. The weakened surface wind stress suggests reduced entrainment of cold water from below the mixed layer. Within 100–200 km of the coast, the typical upwelling‐favorable wind stress curl decreases, suggesting reduced upwelling of cold water. In a 1000‐km area of anomalous warming offshore, the typical downwelling‐favorable wind stress curl also decreases, implying reduced downward Ekman pumping, which would allow mixed‐layer shoaling and amplify the effect of the positive climatological summertime net surface heat flux.

## Key Points

- Extreme warm anomalies on time scales of 10 days to 6 months occur mostly in December through March
- The net surface heat flux anomalies do not explain most of the anomalous warming even when allowing for uncertainty in mixed layer depth
- Wind stress and stress curl weaken in the warming area suggesting reduced entrainment and Ekman pumping and perhaps mixed‐layer shoaling

## 1 Introduction

### 1.1 Marine Heat Waves in the Chile‐Peru Current System and California Current System

Marine heat waves (MHWs) are periods of unusually warm sea surface temperatures (SSTs), or warm anomalies, that occur on time scales of days to months (Hobday et al., 2018; Oliver et al., 2021). MHWs in eastern boundary upwelling systems (EBUSs), such as the Chile‐Peru Current System (CPCS) in the southeast Pacific and the California Current System (CCS) in the northeast Pacific, have the potential to make surface waters too hot for typical local fish populations and the larvae that will become the stock in future years (Cheung & Frölicher, 2020). Fish that do not perish during MHW events may migrate to cooler waters far away, as resulted from the 2014 to 2016 MHW in the CCS (Auth et al., 2018; Bond et al., 2015; Cavole et al., 2016; Daly et al., 2017). Further, high SST anomaly events such as MHWs are associated with reduced populations of copepods and microphytoplankton, threatening dependent fisheries, including in the southeast Pacific Ocean (Iriarte & González, 2004) and CPCS, similar to the 2014–2016 MHW that altered biological activity in the CCS (Cavole et al., 2016; Du & Peterson, 2018; McCabe et al., 2016; Peterson et al., 2017; Whitney, 2015).

**Figure 1:**
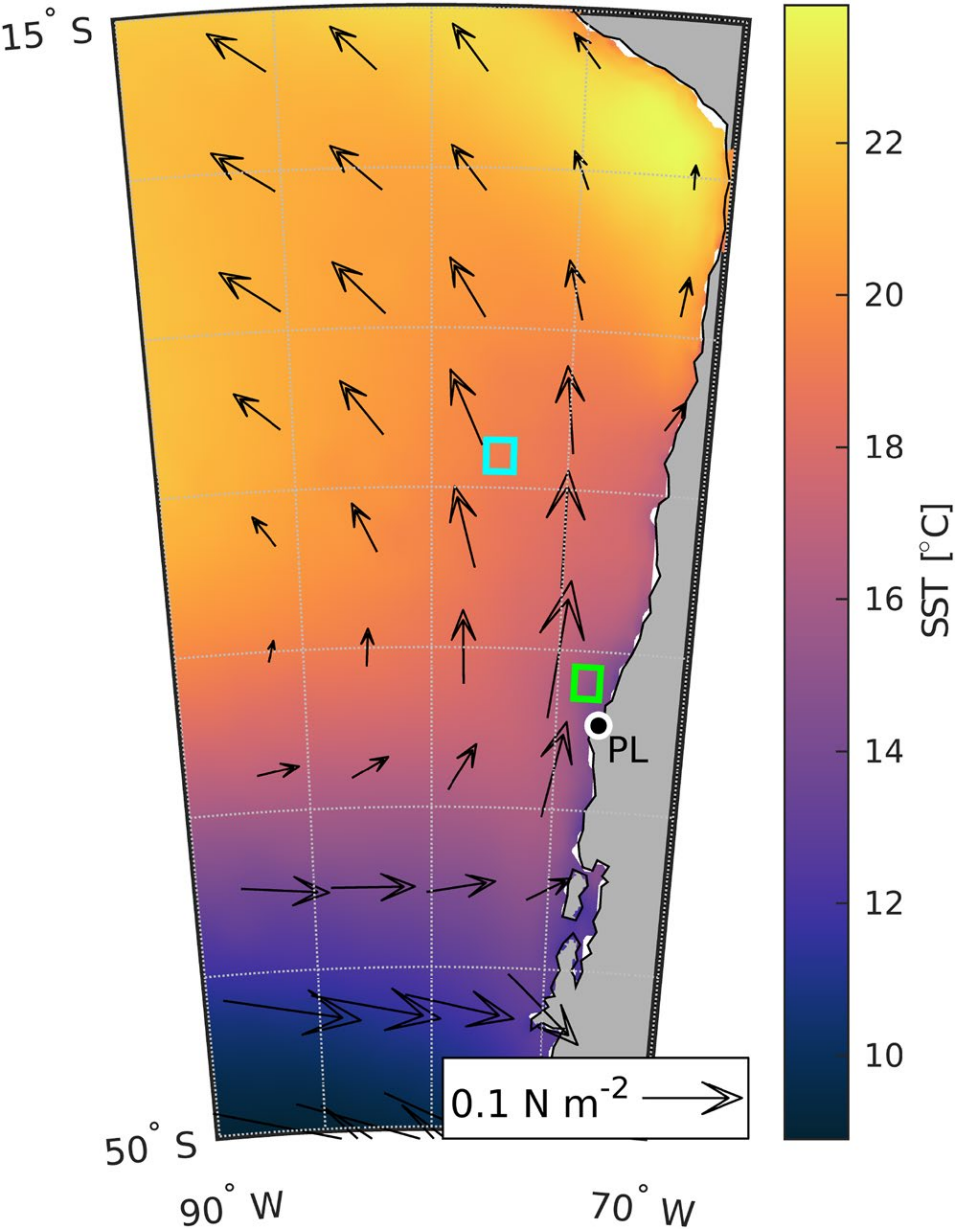
Mean summer sea surface temperature (SST) and wind stress along and offshore of the Chile‐Peru Current System from ERA5. Arrows: mean wind stress during austral summer (December–February). Color shading: mean summer SST. Green box: the area used below to define SST anomaly events (Section 2.6). Cyan box: the area used for the offshore spatially averaged time series described in Section 2.6. The location of Punta Lavapié is indicated by the black dot enclosed by a white circle and labeled “PL”.

The CPCS is the most productive EBUS in the world based on fish harvested per unit area (Montecino & Lange, 2009). The prevailing oceanic flow pattern along the CPCS includes an equatorward jet that develops in the austral spring and summer (Strub et al., 2019). This jet is close to the coast south of the Punta Lavapié headland (Aguirre et al., 2012) (black dot in Figure 1) and the topography then steers the jet offshore as it passes the cape (Mesias et al., 2003). This flow pattern is similar to the separating upwelling jet around Cape Blanco in the CCS (Barth et al., 2000). East of the equatorward near‐surface flow, the pycnocline reaches a relatively shallow depth of 50 m, which allows chlorophyll‐*a* concentrations to remain relatively high near the shore through the winter (Letelier et al., 2009). The offshore meander of the flow northwest of Punta Lavapié pushes the shallow pycnocline and associated front further offshore to extend the section of high‐chlorophyll water (Letelier et al., 2009). Wind stress curl is the dominant driver of the upwelling circulation (Aguirre et al., 2012), and there is less meandering of the jet north of Punta Lavapié during periods of wind relaxation (Mesias et al., 2003). Wind relaxations along the CPCS can be associated with warm water anomalies (Garreaud et al., 2011).

An important component of protecting the natural resources of the CPCS is long‐term monitoring and comprehension of the processes that drive anomalous environmental variability, such as the ocean temperature extremes that are the subject of this analysis. The forcing mechanisms that cause extremely warm SST anomaly events in the subtropical southeast Pacific, along and offshore of the Chile‐Peru EBUS, are not well understood. Currently, there is not enough buoy coverage in the CPCS to track increasing surface temperatures in situ as warm anomaly events develop (Garreaud et al., 2011). The intensity and frequency of extreme ocean temperatures in the eastern Pacific are altered by background ocean conditions from the El Niño/Southern Oscillation (ENSO) and other low‐frequency oscillations (Holbrook et al., 2019). Particularly prolonged MHWs along the CPCS in 2016–2017 were associated with a “coastal” El Niño (Echevin et al., 2018; Garreaud, 2018a, 2018b; Pujol et al., 2022; Rodríguez‐Morata et al., 2019). In the northern CPCS, between the equator and 25°S, MHWs on time scales of a month and longer are typically associated with anomalies of equatorial origin, and MHWs on time scales of ∼10 days and shorter are associated with weakening of local winds (Pietri et al., 2021). In the central and southern CPCS, MHWs have increased in intensity and frequency from 1982 to present (Pujol et al., 2022). However, the central CPCS, from ∼29°S to 38°S, including the Punta Lavapié upwelling center and the area to the north of Punta Lavapié, has experienced different timing and intensities of MHWs than areas farther to the south. The central CPCS exhibited a delayed onset of warming during the 2016–2017 MHWs compared to areas south of 38°S, and the central CPCS has weaker trends in MHW occurrence over the past several decades than the southern CPCS (Pujol et al., 2022). In this study, we focus on the central CPCS region and the potential role of wind anomalies in creating extreme warm SST anomalies on time scales shorter than the 2016–2017 El Niño event, since that event has been addressed in prior studies.

### 1.2 Lessons From Warm SST Events and Wind Relaxations in the CCS

The CCS and CPCS, that is, the EBUSs of the northeast and southeast Pacific, may be thought of as analogous systems. As mentioned in Section 1.1, wind relaxations in the CPCS are observed to be associated with warm SST events (Garreaud et al., 2011). Therefore, studies of wind relaxations and associated SST anomaly patterns in the CCS informed our approach for characterizing warming during wind relaxations in the CPCS. In the CCS, propagating atmospheric cyclones weaken upwelling favorable winds in the summer months of May through August, leading to wind relaxations and intensifications (Fewings et al., 2016; Halliwell & Allen, 1987) with a quasi‐dipole pattern (Fewings, 2017) and associated SST anomalies (Flynn et al., 2017). Composite averages of a surface mixed‐layer anomaly heat budget over many repetitions of the wind relaxation event cycle described in Fewings et al. (2016) revealed clusters of SST anomalies that divided the CCS into northern and southern regions (Flynn et al., 2017). During wind relaxation events in the northern (poleward) half of the CCS, the net surface heat flux, especially the latent heat flux, was the dominant contributor to formation of positive SST anomalies (Flynn et al., 2017). In contrast, during the wind relaxation phase in the southern (equatorward) region of the CCS, air‐sea heat flux anomalies did not explain the observed changes in SST during the wind relaxation events. Even so, the SST anomalies increased with time during the wind relaxations south of Cape Mendocino (Flynn et al., 2017, their Figure 8c, day 5). Flynn et al. (2017) proposed that the warming during these wind relaxation events was most likely caused by decreased entrainment and vertical Ekman pumping at the base of the mixed layer, and, in the California Current extension region, reduced advection of cold water from farther north.

In July 2015, during the 2014–2016 MHW in the CCS, a strong positive SST anomaly and associated wind stress anomaly extended southwest from Cape Mendocino (Fewings & Brown, 2019), a known upwelling center (Largier et al., 1993). During that event, a longer than average southern wind relaxation event prolonged the warming conditions so that the spatial patterns of the SST anomaly were similar to that of the wind stress anomaly (Fewings & Brown, 2019). During more common shorter southern wind relaxation events in the CCS, the wind stress anomaly had a more complicated relationship to the evolution of the SST anomaly field. Since SST was preconditioned to be cooler during these events on average (Flynn et al., 2017), due to a preceding phase of the wind event cycle, the wind stress anomaly exhibited a strong spatial correlation with temporal changes in the SST anomaly field, rather than the SST anomaly itself. Therefore, it is more informative to look at the relationship between the wind stress anomalies and the time derivative of SST rather than SST itself.

As mentioned in Section 1.1, the evolution of the wind stress magnitude and wind stress curl strongly influences the upwelling circulation of the CPCS. The wind direction along the CPCS is predominantly equatorward (Figure 1) and the strength of alongshore wind stress in this direction primarily determines the strength of coastal upwelling (Bakun & Nelson, 1991). Numerical simulations have revealed how upwelling‐favorable wind stress in the region is dominated by signals with periods of 20 days or longer (Mesias et al., 2003). West to east propagating anticyclones form coastal lows at 30°S over the coast of Chile such that the winds relax or reverse to flow offshore around 40°S while the coastal lows evolve (Garreaud et al., 2002), analogous to the wind relaxations in the CCS. A historical reanalysis provided a benchmark in a study of propagating anticyclones in EBUS for comparison with climate projections, which predict that the paths of these anticyclones will shift poleward (Aguirre et al., 2019). The Chilean Upwelling Experiment (CUpEx) off north‐central Chile also documented a stable southerly wind regime and warming of 0.5°C–1°C per day during weak or reversed winds (Garreaud et al., 2011). Our study region includes areas south of the CUpEx study area, areas known to have more frequent weather systems pass along the mid‐latitude storm track south of 30°S, some of which cause the wind relaxations discussed above (Garreaud et al., 2011).

**Figure 2:**
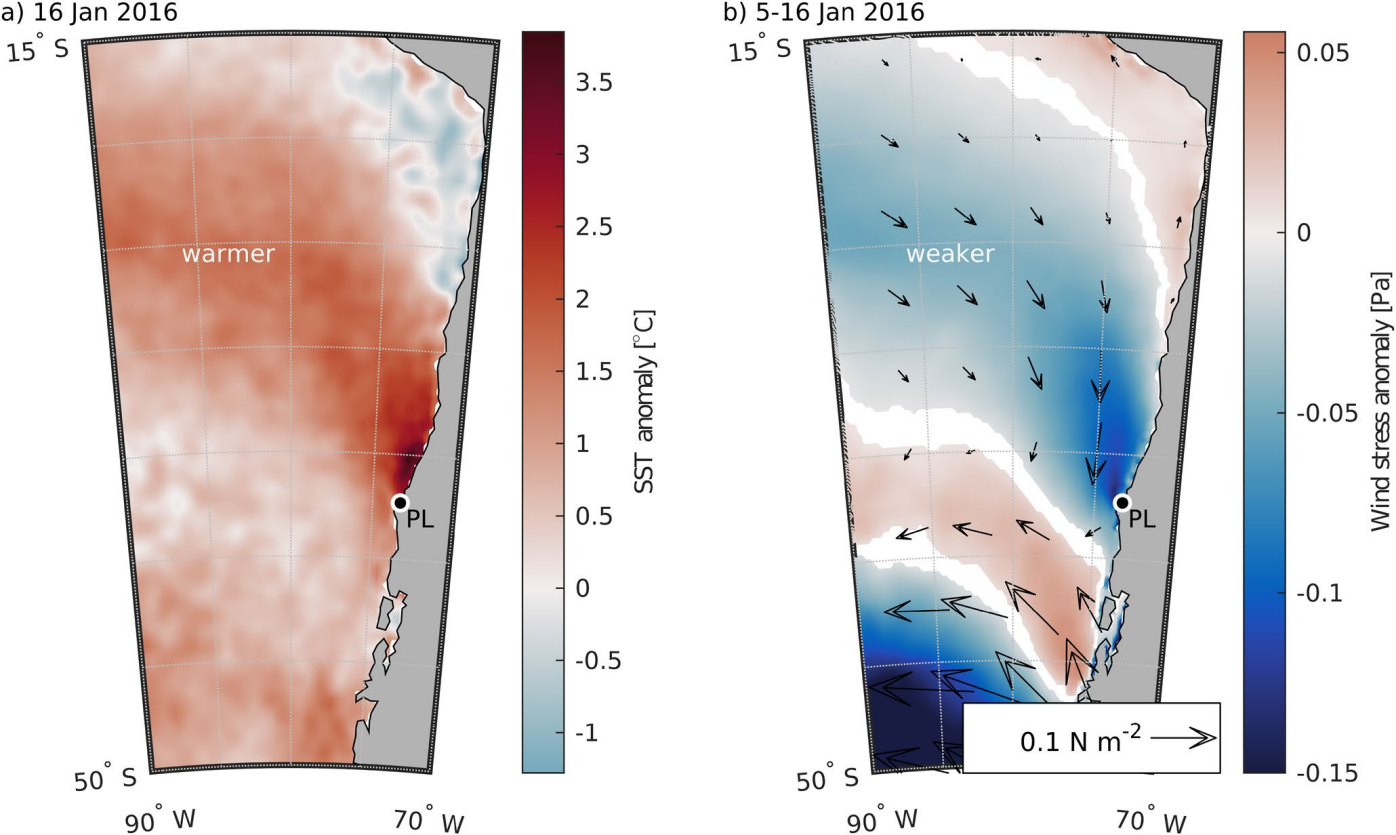
A January 2016 warm sea surface temperature (SST) anomaly and preceding wind stress anomaly. (a) Daily SST anomaly off western South America on 16 January 2016, relative to the daily climatology during 1979–2020, from ERA5. (b) Color shading: mean wind stress anomaly during 5–16 January 2016 from ERA5, calculated from daily averages of the ERA5 accumulated hourly surface wind stress magnitude anomaly and arrows: vector wind stress anomalies, relative to the climatological mean for 5 January 1979–16 January 2020. White areas indicate where the mean wind stress anomaly during 5–16 January 2016 was not outside the 95% confidence interval on the climatology, that is, the anomaly was not different from zero by more than the uncertainty in the climatology. In each panel, Punta Lavapié is indicated by the black dot enclosed by a white circle and labeled “PL”.

An example of an extreme warm event and associated wind relaxation offshore of the CPCS occurred in January 2016. Remotely sensed unfiltered SST anomalies in the CPCS reveal a significant warm SST anomaly event in mid‐January (Figure 2). The warmest daily SST anomalies (Figure 2a) were at least 3°C, and SST anomalies in this area were paired with weakened wind stresses (relaxation) (Figure 2b). Both the positive SST anomaly and negative wind stress anomaly extended offshore to the northwest from the Punta Lavapié upwelling center near the coast. This wind pattern over the CPCS is qualitatively similar to wind relaxations over the CCS and occurs in response to the atmospheric subtropical high either weakening or moving further west (e.g., Jiang et al., 2010). In this study, we analyze a suite of similar events.

### 1.3 The Ocean Surface Mixed Layer Heat Budget as a Tool

In previous studies, an anomaly heat budget for the ocean surface mixed layer has been a useful tool to determine whether observed SST anomalies can be explained by air–sea heat flux anomalies or must be explained by other processes. A surface mixed‐layer anomaly heat budget is derived from the conservation of mass and heat equations to relate the transfer of heat to SSTs (Stevenson & Niiler, 1983). Changes in SST are used as a proxy for the changing heat content in the ocean surface mixed layer, and these changes can be compared at a particular time by using the differential form of the heat budget equation, as in this study, or over a period of time by using the integral form, as for the CCS in Flynn et al. (2017) and Fewings and Brown (2019). Observations of the net surface heat flux anomaly, mixed‐layer depth (MLD), temperature gradients, vertical mixing, advection, and eddy diffusivity allow us to estimate the scale of terms in the heat budget equation, such that the terms that are less significant to the change in heat content may be neglected (Stevenson & Niiler, 1983). Holbrook et al. (2019) compared MHWs globally with an upper ocean mixed‐layer heat budget to identify important regional processes, ocean and atmosphere teleconnections, and large‐scale climate modes. Among regional processes, the net surface heat flux anomaly was small and advective terms were likely negligible more than several hundred km offshore in the CCS (Correa‐Ramirez et al., 2007; Flynn et al., 2017), so Flynn et al. (2017) inferred from the wind field evolution that mixed layer temperature changes were forced by decreased vertical entrainment and mixed layer shoaling, as mentioned above.

### 1.4 Research Questions

The goal of this analysis was to identify the regional drivers of extreme warm SST anomalies along and offshore of the CPCS, focusing on time scales shorter than previously studied El Niño events, and to compare and contrast these warm events with the causes of events studied previously along and offshore of the CCS. Due to the biological significance of the Punta Lavapié upwelling center as a food and bait source, we limited the focus of this study to extreme warm events affecting that area. We used the surface mixed‐layer anomaly heat budget to answer the following research questions:

1. Do historical warm SST anomaly events on time scales of 10 days to 6 months and the associated areas of maximum warming affecting Punta Lavapié in the CPCS have a common spatial pattern and offshore extent?
2. Can the net surface heat flux anomaly account for most of the anomalous warming during these events?
3. Does the spatial pattern of anomalous warming coincide with a weak wind stress anomaly pattern, or changes in wind stress curl, as in the case of warming SST following wind relaxations in the CCS?

As we analyzed data to answer research question 2, we used two approaches with different approximations of MLD. These approaches were designed to answer the following sub‐questions:

2a. Can a fixed MLD based on a regional climatology from Argo profiles, combined with observations of the net surface heat flux anomaly, explain all of the anomalous warming?
2b. What MLD would be required in our study area if all anomalous warming were driven by the net surface heat flux anomaly, and how does that hypothetical MLD compare with the typical observed summer MLD?

## 2 Data and Methods

### 2.1 Data

SST, surface wind stress, and surface heat flux data were retrieved from the fifth generation European Centre for Medium‐Range Weather Forecasts (ECMWF) Reanalysis (ERA5) (Hersbach et al., 2018). We retrieved data from 1979 to 2020 on a latitude‐longitude grid with 0.25° grid spacing for the southeast Pacific from 15°S to 50°S and 70°W to 90°W. The SST from ERA5 is a daily mean value. We estimated the rate of warming, or partial time derivative of SST, from the daily SST values using the centered difference approximation. For the northward and eastward components of the surface wind stress, and for the components of the net surface heat flux (Section 2.4), we obtained accumulated hourly values from the single level sea surface data set of ERA5 and then averaged the accumulated hourly values over each day.

To characterize wind stress and wind stress curl variability associated with warm SST events, we additionally used Level 2 (L2) satellite scatterometer winds from QuikSCAT (SeaPAC, 2020) and from the Advanced Scatterometer on the MetOp‐A satellite (ASCAT‐A). To form the climatologies and anomalies, for each scatterometer data set we extracted a time period consisting of complete years. For QuikSCAT, we used data from 1 November 1999 to 30 October 2009. Two versions of ASCAT‐A were used for this study: (a) the KNMI ASCAT‐A 25‐km product (EUMETSAT/OSI SAF, 2010b; Verspeek et al., 2010) from 1 June 2007 to 31 May 2021 and (b) the KNMI ASCAT‐A 12 km coastal‐optimized product (EUMETSAT/OSI SAF, 2010a; Verhoef & Stoffelen, 2013) from 1 September 2010 to 31 August 2021. The ASCAT‐A coastal product is optimized to provide wind retrievals closer to the coast, but it is not currently publicly available before 2010. As we show later, the wind stress curl signature associated with the warming events is strong within ∼100 km of the coast and is not well captured by the ASCAT‐A 25‐km data set. Vector wind stresses were computed from the L2 scatterometer 10‐m equivalent neutral winds using the stress formulation from the COARE v3.0 bulk flux algorithm (Fairall et al., 2003) as implemented in (O’Neill et al., 2012). The L2 wind stresses were constructed onto a uniform 0.25° latitude‐longitude grid and the wind stress curl was computed from the gridded swath‐level wind stress vectors.

### 2.2 Calculating Wind Stress Magnitude

Because previous analyses of anomalously warm events in the CCS have noted that mixed layer shoaling could amplify the warming from the net surface heat flux (Fewings & Brown, 2019; Flynn et al., 2017), and because weakened winds, regardless of wind direction, may contribute to mixed layer shoaling through reduced shear‐driven mixing (Price et al., 1986), we calculated the surface wind stress magnitude. The surface wind stress magnitude was calculated from the ERA5 eastward and northward components of the hourly accumulated wind stress, *τ*_*x*_ and *τ*_*y*_, and then averaged to get the daily mean wind stress magnitude $\left(\left|\vec{\tau }\right|\right)$.

### 2.3 Calculating Daily Anomalies

At each grid point, we calculated a climatological daily value by sorting ERA5 daily values (for SST) or our daily averages (for other variables) from 1 January 1979 to 31 December 2020 by day of the year and then analyzed the average for each day of the year. Then we computed daily anomalies for the entire 1979–2020 time series by subtracting the climatological value for a given calendar day from the observed value. This process was applied to each location for the time series of *SST*, *∂SST*/*∂t*, the components of the net surface heat flux *Q*_*net*_ (Section 2.4), and the daily average wind stress magnitude $\left|\vec{\tau }\right|$. The daily anomalies computed in this way are denoted by primes hereafter as *SST*′, *∂SST*′/*∂t*, the components of $Q_{\mathrm{net}}^{\prime }$, and $|\vec{\tau }{|}^{\prime }$.

For each of the three wind stress curl satellite products, we calculated a separate annual climatology for each data set's period of record (Section 2.1) using the same method as for the ERA5 annual climatologies above. We then calculated the daily anomalies $\nabla \times {\vec{\tau }}^{\prime }$ for each of the three wind stress curl data sets by evaluating the difference between the original data set and the annual climatology for each day of the year.

### 2.4 Estimating Net Surface Heat Flux Anomalies

The net surface heat flux anomaly $Q_{\mathrm{net}}^{\prime }$ is the sum of the anomalies of the four components of the surface heat flux into the ocean: the anomalous net shortwave radiation $\left(Q_{\mathrm{SWR}}^{\prime }\right)$, anomalous net longwave radiation $\left(Q_{\mathrm{LWR}}^{\prime }\right)$, sensible heat flux anomalies $\left(Q_{\mathrm{SHF}}^{\prime }\right)$, and latent heat flux anomalies $\left(Q_{\mathrm{LHF}}^{\prime }\right)$:

(1) $$Q_{\mathrm{net}}^{\prime }=Q_{\mathrm{SWR}}^{\prime }+Q_{\mathrm{LWR}}^{\prime }+Q_{\mathrm{SHF}}^{\prime }+Q_{\mathrm{LHF}}^{\prime }\;.$$

The sign convention used here is that the surface heat flux *Q*_*net*_ is positive when heat is transferred to the ocean surface mixed layer through the air‐sea interface. Therefore, the surface heat flux anomaly $Q_{\mathrm{net}}^{\prime }$ is positive when more heat is added to the ocean surface mixed‐layer than usual, that is, more than in the climatology for that day of the year.

### 2.5 Filtering

Other studies have focused on ENSO influences on the CPCS (Section 1.1). Here, in order to focus on warm anomalies associated with regional processes, we band‐pass filtered the data to focus on events with time scales between 10 days and 6 months. This removes temporal variability associated with ENSO or other long time scale, large‐scale warming processes distinct from the warm SST events of interest in this study. By restricting this study to events with time scales longer than 10 days, rather than five days as in the Hobday et al. (2016) definition of MHWs, the anomalously warm events in this study are more comparable with similar extreme events in the CCS such as the July 2015 event, which lasted multiple weeks (Fewings & Brown, 2019). Since our events do not necessarily meet the widely used Hobday et al. (2016) definition of MHWs, we refer to these events as warm SST anomaly events, anomalously warm events, or variations of this. Additionally, removing the variability on time scales longer than 6 months allows us to maintain our focus on events that we can compare to previous studies of wind relaxation events in the CCS.

The temporal band‐pass filter was applied to the daily anomalies of *SST*′, *∂SST*′/*∂t*, $Q_{\mathrm{net}}^{\prime }$, and the wind stress magnitude anomaly. We applied the low‐pass filter PL66 (Beardsley et al., 1985) twice to isolate signals occurring on time scales between 10 days and 6 months. In the time domain, PL66 is a piecewise parabolic and linear weighting function, giving the transfer function a sharp frequency cutoff and smaller and narrower side lobes than a Lanczos filter (Beardsley et al., 1985). First, we applied PL66 to the daily average data, using a half amplitude cutoff frequency *f*_0_ = 1.16 × 10^−6^ Hz, or 1 cycle per 10 days. Second, we applied PL66 to the once‐filtered daily average data again, but using a half‐amplitude cutoff frequency *f*_0_ = 6.34 × 10^−8^ Hz, or 1 cycle per 6 months. By subtracting the second time series from the first time series, we created the band‐pass‐filtered signal. After removing two window lengths of 6 months from each end to avoid edge effects, this data set spans the period of January 1980 through the end of December 2019.

### 2.6 Defining Warm Events and Associated Warming Events

**Figure 3:**
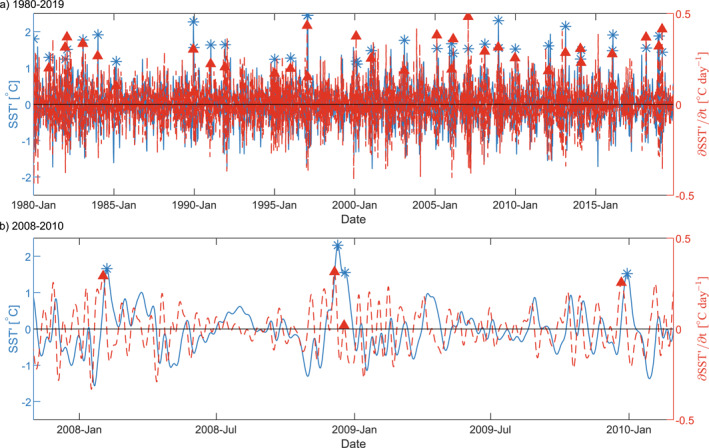
Time series of sea surface temperature (SST) anomaly and its time derivative in the region used to define events. (a) 10‐day to 6‐month band‐pass‐filtered SST anomaly *SST*′ (blue time series) and rate of change of SST anomaly $\frac{\partial SST^{\prime }}{\partial t}$ (red time series) from ERA5, spatially averaged over the green square in Figure 1, ∼100 km offshore of the Punta Lavapié upwelling center. Blue stars indicate times of the 37 extreme warm events and red triangles indicate the 37 associated times of warming events as defined in Section 2.6. (b) A section of the time series from (a) including January 2008 to January 2010.

We defined warm SST anomaly events based on daily SST anomalies in the area offshore of Punta Lavapié. To find warm events, we used a spatial average of the *SST*′ time series within a 1° by 1° area approximately 50–150 km offshore (green box in Figure 1). Although this spatial average is taken within the zone that can be influenced by filaments of recently upwelled water, the events found in this time series were very similar in timing to the set of events found when we used a box of the same size 200–300 km offshore to the northwest (cyan box in Figure 1). We defined the times of warm events as the times of peaks in *SST*′ greater than two standard deviations above the climatological annual cycle (Figure 3, blue stars), using the standard deviation of all bandpass‐filtered SST anomalies, such that peaks of *SST*′ must be greater than 2*σ* = 1.1°C. This definition differs from the Hobday et al. (2016) definition where MHWs occur when the unfiltered SST is greater than 90% of the values recorded for that day of the year and the SST remains above this threshold value for at least five consecutive days as the threshold value changes with the climatological SST cycle (Hobday et al., 2016; Oliver et al., 2018).

**Figure 4:**
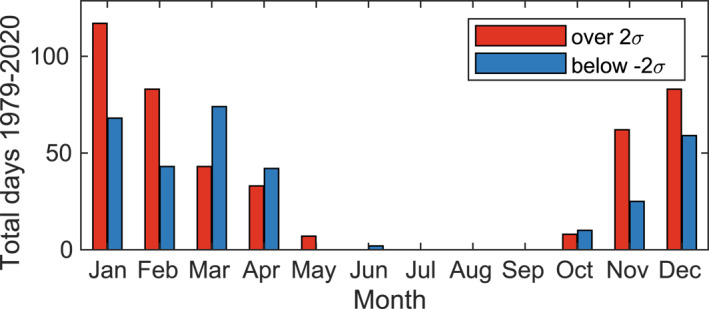
Annual distribution of days with extreme sea surface temperature (SST) anomalies *SST*′ near 36°S off the coast of Chile (green box in Figure 1). Anomalies were filtered to retain time scales between 10 days and 6 months. Only days with SST anomalies that exceeded two standard deviations from zero are included, with positive anomalies shown in red and negative anomalies shown in blue.

In our band‐pass‐filtered SST anomaly time series, most days with extreme positive SST anomalies (over two standard deviations above the mean) off central Chile occur between December and February, the austral summer and upwelling season (Figure 4). For that reason, and to more easily compare warm anomaly events in the CPCS with previously studied warm events in the boreal summer upwelling season in the CCS (Section 1.2), we restricted our analysis to events occurring between December and February. This restricts our number of independent events from 68 to 38 warm events that met these criteria. The annual distribution of warm events (blue stars in Figure 3) in other seasons was: 12 events in spring (September–November), 18 in fall (March–May), 0 in winter (June–August); not shown, but qualitatively related to red bars in Figure 4.

We then defined the *warming* event that preceded each warm event identified above. A similar spatial average in the same nearshore 1° by 1° area but for *∂SST*′/*∂t* was used to identify the nearest time of peak anomalous warming preceding each maximum in *SST*′ (Figure 3, red triangles). Due to the first warm event occurring near the beginning of the band‐pass‐filtered record, there were only 37 times identified of maximum anomalous warming before warm events. Therefore, in the analyses below we use the 37 warming and 37 warm events.

### 2.7 Surface Mixed‐Layer Anomaly Heat Budget

We started with the differential form of the depth‐averaged heat budget for the surface mixed layer, similar to Flynn et al. (2017) and Fewings and Brown (2019):

(2) $$\begin{array}{lll} \frac{\partial SST}{\partial t} & = & \underset{a}{\underset{︸}{\frac{Q_{\mathrm{net}}}{\rho_{w}c_{p}h}}}\underset{b}{\underset{︸}{-\frac{Q_{\mathrm{SWR,}-h}}{\rho_{w}c_{p}h}}}\underset{c}{\underset{︸}{-\bar{\vec{u}}\cdot \nabla_{H}SST}}\underset{d}{\underset{︸}{-\kappa_{H}\nabla_{H}^{2}SST}}\\ & \; & \underset{e}{\underset{︸}{-\frac{\left(SST\;-\;T_{-h}\right)}{h}\left(\frac{\partial h}{\partial t}+{\vec{u}}_{-h}\cdot \nabla_{H}h+w_{-h}\right)}}\underset{f}{\underset{︸}{-\frac{1}{h}\nabla_{H}\cdot \int_{-h}^{0}\tilde{\vec{u}}\;\;\tilde{T}dz}} \end{array}$$

where the left hand side is the rate of change in SST with time *t*. As mentioned previously, and similar to previous studies, SST is used as a proxy for the vertically averaged temperature within the mixed layer. The first term on the right side of Equation 2 is the net surface heat flux *Q*_*net*_ divided by the density of seawater, *ρ*_*w*_, the specific heat capacity of seawater, *c*_*p*_, and the mixed layer depth (MLD), *h*, which converts *Q*_*net*_ into a rate of temperature change. We used values of *ρ*_*w*_ = 1025 kg m^−3^ (Silva et al., 2009; Talley et al., 2011) and *c*_*p*_ = 3850 J kg^−1^ °C^−1^ (Talley et al., 2011). Terms (b)–(f) represent interior ocean processes that affect SST, including: (b) penetrating radiation absorbed below the mixed‐layer, where *Q*_*SWR,−h*_ is the shortwave radiative flux at the base of the mixed layer (depth *z* = −*h*, where *z* = 0 is defined to be at the mean sea surface); (c) horizontal advection of temperature gradients, where $\vec{u}$ is the horizontal velocity, overbar indicates vertical average over the mixed layer, and ∇_*H*_ is the horizontal gradient operator; (d) horizontal eddy diffusion of temperature, where *κ*_*H*_ is a horizontal eddy diffusivity; (e) entrainment at the base of the surface mixed‐layer, where *T*_−*h*_ is the temperature just below the base of the mixed layer and ${\vec{u}}_{-h}$ and *w*_−*h*_ are the horizontal and vertical velocities at the base of the mixed layer, respectively [see Flynn et al. (2017) for more details]; and (f) the covariance between deviations of horizontal velocity and temperature within the mixed layer from their vertical averages within the mixed layer, where tilde (*^*) indicates the vertical average has been removed.

To isolate the influence of the net surface heat flux anomalies on the development of SST anomalies, we simplified Equation 2 to an equation for the change in temperature due to the net surface heat flux only. We retained only term (a) from Equation 2, absorbing the other terms into a residual, and replacing MLD in (a) with its climatological summer value *h*_0_:

(3) $$\frac{\partial SST}{\partial t}=\frac{Q_{\mathrm{net}}}{\rho_{w}c_{p}h_{0}}+R,$$

where the residual *R* contains terms (b)‐(f) from Equation 2 as well as the effects of departures of MLD *h* from the climatological value. Next, by removing the climatology from each term, we formed an anomaly heat budget equation:

(4) $$\frac{\partial SST^{\prime }}{\partial t}=\frac{Q_{\mathrm{net}}^{\prime }}{\rho_{w}c_{p}h_{0}}+R^{\prime }$$

where primes (′) indicate the climatology has been removed.

### 2.8 Compositing Anomalies at Maximum Warming

To understand the cause of high *SST*′ events (blue stars in Figure 3), we examined the surface mixed‐layer anomaly heat budget (Equation 4) at the times of peak anomalous warming before those events (red triangles in Figure 3). First, at each location in the study area, we determined *∂SST*′/*∂t* at the time of peak warming before each of the 37 events (red triangles in Figure 3). Next, at each location, we calculated a composite average of *∂SST*′/*∂t* over those 37 times of peak anomalous warming. By mapping the composite averages, we determined the spatial extent of maximum *∂SST*′/*∂t* for the composite mean event.

The 95% confidence interval on a mean at a given location is defined by (Bendat & Piersol, 1986)

(5) $$\mu_{y}={\hat{\mu }}_{y}\pm \delta {\hat{\mu }}_{y}\text{, with}\;\delta {\hat{\mu }}_{y}=\frac{{\hat{\sigma }}_{y}}{\sqrt{N}}q_{t}\left(\alpha /2,N-1\right)$$

where *μ*_*y*_ is the true mean, ${\hat{\mu }}_{y}$ is the sample estimate of the mean, and $\delta {\hat{\mu }}_{y}$ is the uncertainty in the sample estimate. In the uncertainty, ${\hat{\sigma }}_{y}$ is the sample estimate of the standard deviation, *α* = 0.05 because we are interested in the 95% significance level, *q*_*t*_(*α*/2, *N* − 1) is the upper tail of a Student‐t distribution at the *α*/2 point with *N* − 1 degrees of freedom, and *N* is the number of degrees of freedom, which here is equal to 37 for the number of independent events. When mapping the composite anomalies below, we excluded areas where the 95% confidence interval on the composite mean anomaly (i.e., ${\hat{\mu }}_{y}\pm \delta {\hat{\mu }}_{y}$) includes zero.

A similar composite average and confidence interval was evaluated for the other anomalies calculated in Section 2.3. The anomalous warming from the $Q_{\mathrm{net}}^{\prime }$ term in the anomaly heat budget (Equation 4) was averaged at the time of peak anomalous warming *∂SST*′/*∂t* before each of the 37 events (Figure 3, red triangles). The difference between the composite average of *∂SST*′/*∂t* and the composite average of the $Q_{\mathrm{net}}^{\prime }/\rho_{w}c_{p}h_{0}$ term yielded the estimate of the composite mean residual *R*′ over the 37 events as in Equation 4. The difference between the quantities *∂SST*′/*∂t* and $Q_{\mathrm{net}}^{\prime }/\rho_{w}c_{p}h_{0}$ for individual events was used to find a standard deviation and 95% confidence interval for the residual temperature change *R*′, similarly to Equation 5. Then, to estimate the mean surface wind stress magnitude anomaly at times of maximum anomalous warming, the same process was used to calculate the composite average and 95% confidence interval of the surface wind stress magnitude anomalies (Section 2.2). Similarly, we calculated a composite average of *SST*′ at the time of the warm events (blue stars in Figure 3).

We also computed a composite average for the wind stress curl anomalies at the time of peak warming. For each of the three satellite wind stress products, we averaged the wind stress curl anomalies at the times of peak warming (red triangles in Figure 3) that occurred when that product was available. In this case, when evaluating the 95% confidence interval bounds in Equation 5, the number of observations, *N*, in the confidence interval was the number of our events that fell within the period of record of the scatterometer product. For comparison, we also calculated the austral summer mean wind stress curl pattern for each scatterometer product by averaging all daily wind stress curl values that occurred in December, January, or February.

To convert from wind stress curl anomalies to the vertical Ekman pumping velocity anomaly $w_{Ek}^{\prime }$, we applied the following calculation as a function of latitude:

(6) $$w_{Ek}^{\prime }=\frac{\nabla \times {\vec{\tau }}^{\prime }}{\rho_{w}f}\;\text{with}\;f=2\Omega \sin\;\theta$$

as in Kraus and Businger (1994) and Flynn et al. (2017). In Equation 6, $\nabla \times {\vec{\tau }}^{\prime }$ is the curl of the wind stress vector anomaly described in Section 2.3, *f* is the Coriolis parameter, Ω is the rate of angular rotation of the Earth, and *θ* is the latitude in degrees.

### 2.9 Mixed‐Layer Depth Climatology

Our estimate of the contribution of the $Q_{\mathrm{net}}^{\prime }$ term to the rate of anomalous warming in Equation 4 depends on the value of the climatological MLD *h*_0_. We used an estimate of *h*_0_ = 25 m based on a seasonal MLD climatology from Argo float profiles. We began with the monthly climatological MLD values as in Holte et al. (2017) provided at http://mixedlayer.ucsd.edu. These monthly climatologies contain missing values when too few Argo profiles were available within a grid cell. We calculated the summer mean climatological MLD in our study region by averaging the monthly MLD climatologies from Holte et al. (2017) over the months of December, January, and February at each location. In this step, locations where the MLD for one or more months was missing were also left missing in the summer mean MLD. This ensured that for a summer mean MLD, we would not consider any mean values where an insufficient number of profiles were sampled for one or more of the months, which could cause a bias in the summer mean estimate. The total number of floats per location and standard deviation of the MLD provided with the monthly climatologies from Holte et al. (2017) were used in the 95% confidence interval on an overall mean.

### 2.10 Linear Regression for MLD Assuming No Residual

To test the possibility that the net surface heat flux anomaly could explain all anomalous warming, we calculated a hypothetical best‐fit MLD for a scenario where the residual in Equation 4 equals zero. For that scenario, we rewrote Equation 4 as $\frac{Q_{\mathrm{net}}^{\prime }}{\rho_{w}c_{p}}=h_{0}\frac{\partial SST^{\prime }}{\partial t}$. First, we calculated the correlation coefficient between *∂SST*′/*∂t* and $Q_{\mathrm{net}}^{\prime }/\rho_{w}c_{p}$ for the 37 events at each location to determine where in the study domain a linear relationship between those terms was statistically significant. Then we used linear regression to fit the following model:

(7) $$\frac{Q_{\mathrm{net}}^{\prime }}{\rho_{w}c_{p}}=\hat{h}\;\frac{\partial SST^{\prime }}{\partial t}+\epsilon ,$$

where the observed $Q_{\mathrm{net}}^{\prime }/\rho_{w}c_{p}$ is modeled as a linear function of the observed *∂SST*′/*∂t*, $\hat{h}$ is the best‐fit coefficient of the linear term which defines the best fit line, and *ϵ* is the error in the model. This linear coefficient $\hat{h}$ is the MLD that is consistent with the case where $Q_{\mathrm{net}}^{\prime }$ is responsible for all mixed‐layer warming preceding the warm events. For each location, we calculated the linear slope coefficient $\hat{h}$ from this regression using the 37 events.

At each location, we also tested whether the skill $\hat{S}$ of the model in Equation 7 was greater than the critical skill ${\hat{S}}_{\mathrm{crit}}$, assuming a Gaussian distribution for *N* = 37 degrees of freedom. The equations for these are

(8) $$\hat{S}=\frac{{\hat{\sigma }}_{\hat{y}}^{2}}{{\hat{\sigma }}_{y}^{2}}$$

(9) $${\hat{S}}_{\mathrm{crit}}\left(\alpha ,1,N\right)=\frac{q_{F}\left(\alpha ,1,N-2\right)}{\left(N-2\right)+q_{F}\left(\alpha ,1,N-2\right)},$$

where $\hat{S}$ is the skill of the model at a location, ${\hat{\sigma }}_{\hat{y}}^{2}$ is the sample variance of the linear regression model, and ${\hat{\sigma }}_{y}^{2}$ is the sample variance of the observations (Emery & Thomson, 2001). For the null hypothesis test, ${\hat{S}}_{\mathrm{crit}}$ is the critical skill level, *α* = 0.05 is the significance level, *N* = 37 is the number of degrees of freedom, and *q*_*F*_(*α*, 1, *N* − 2) is the upper tail of the Fisher F‐distribution for a univariate linear regression (Emery & Thomson, 2001). At locations where $\hat{S}<{\hat{S}}_{\mathrm{crit}}$, we do not report a MLD estimate $\hat{h}$ from the linear regression model.

## 3 Results

### 3.1 Spatial Pattern of Anomalous Warm Events and Warming Events

**Figure 5:**
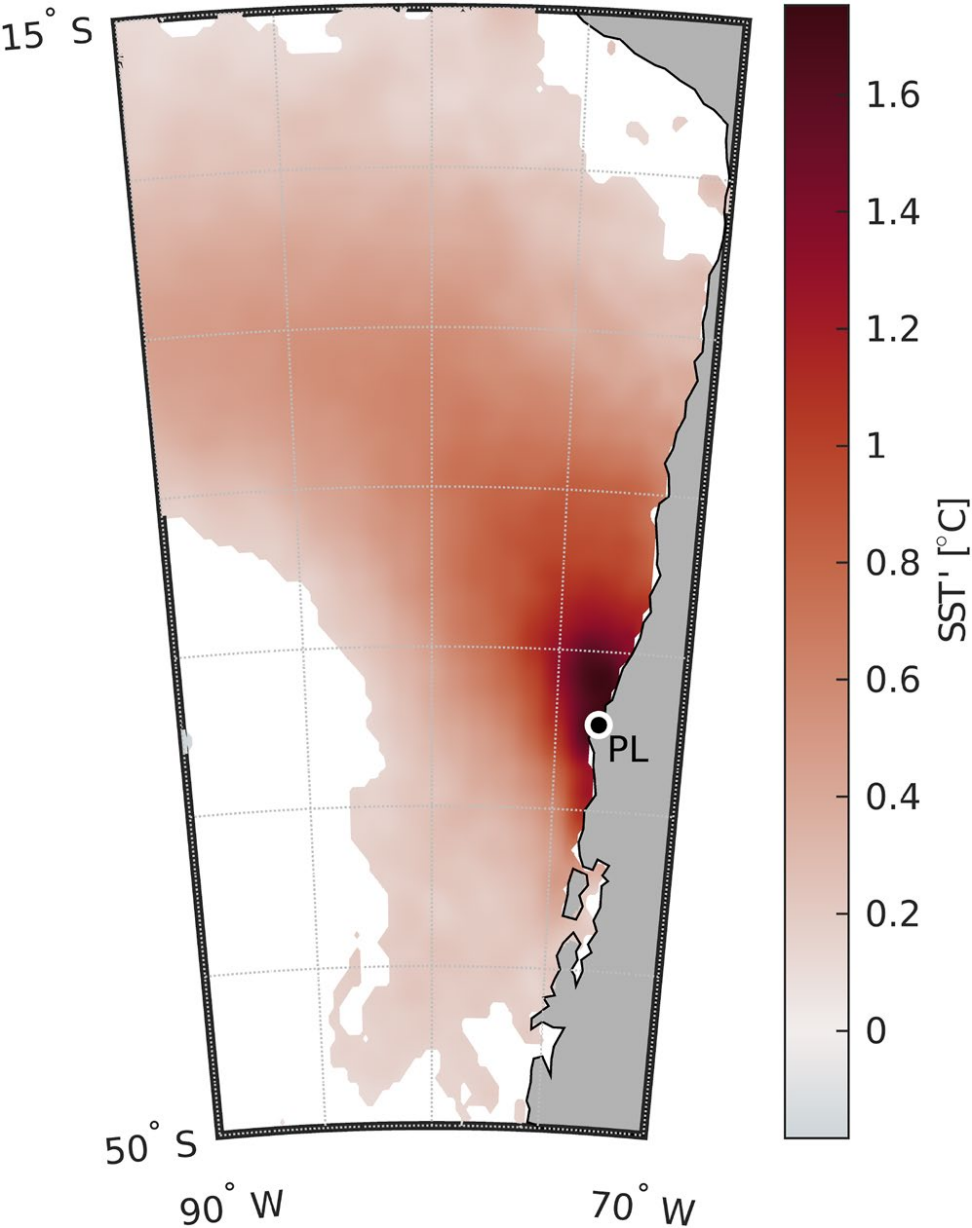
Composite average sea surface temperature (SST) anomaly *SST*′ over 37 warm events (blue stars in Figure 3). White indicates areas where the composite mean anomaly is not significantly different from zero at the 95% confidence level. *SST*′ was band‐pass filtered to retain temporal variability with time scales from 10 days to 6 months. Punta Lavapié is indicated by the black dot enclosed by a white circle and labeled “PL”.

The example warm anomaly event in January 2016 in the CPCS (Section 1) motivated our analysis of other historical warm SST anomaly events in the same area. To determine whether the 37 historical extreme warm SST events (blue stars in Figure 3) had a consistent spatial pattern, we examined the composite average *SST*′ over the 37 warm events. The area of anomalously warm SST was qualitatively similar to the January 2016 event (compare Figures 2a and Figure 5). The highest SST anomalies, over 1.6°C, tend to be localized near the coast north of Punta Lavapié (Figure 5). In contrast, the highest offshore warm anomalies are about half as warm, for example, 0.7°C along 80°W between 15°S and 50°S.

**Figure 6:**
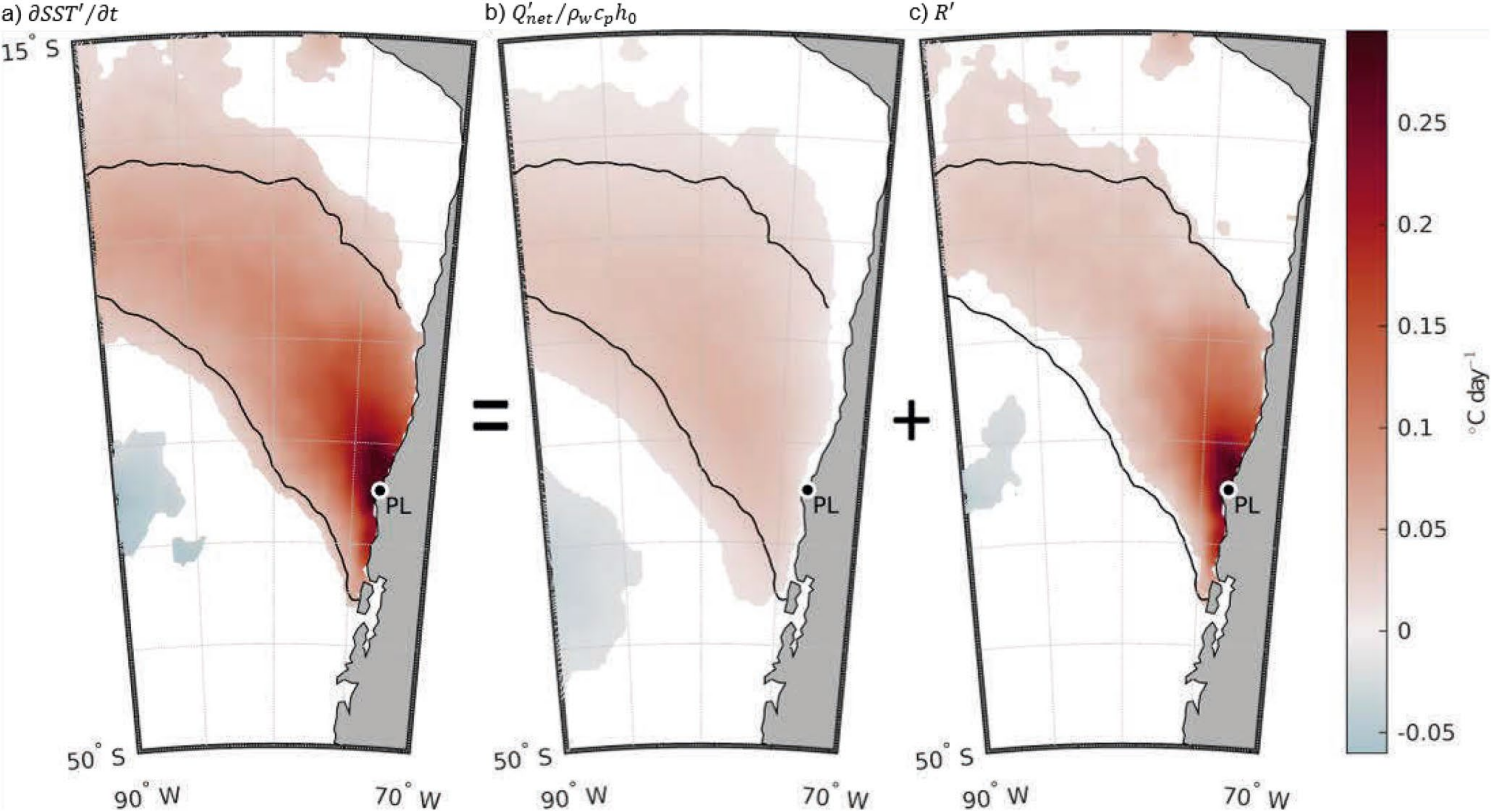
Terms in the anomaly heat budget. (a) The composite mean of the anomalous warming, *∂SST*′/*∂t*, composited over the 37 events. As in Equation 4, (a) equals the sum of (b) the composite mean warming from the anomalous air‐sea heat flux term $Q_{\mathrm{net}}^{\prime }/\rho_{w}c_{p}h_{0}$ and (c) the residual temperature change *R*′. White in each panel indicates areas where the composite mean is not significantly different from zero at the 95% confidence level. The black contour is the same in each panel and encloses the area where substantial anomalous warming is observed, *∂SST*′/*∂t* ≥ 0.05°C dy^−1^. In each panel, Punta Lavapié is indicated by the black dot enclosed by a white circle and labeled “PL”.

Next, we examined the spatial pattern of warming, *∂SST*′/*∂t*, preceding those warm events offshore of the Punta Lavapié upwelling center. Based on the spatial similarities between the wind stress anomaly and SST anomaly in the January 2016 event (Figure 2), and the link previously shown between wind stress anomalies and warming SST in the CCS (Section 1.2), we hypothesized the pattern of anomalous warming would be a band reaching offshore and toward the equator from the upwelling center, similar to the spatial pattern of the January 2016 warm SST anomaly. Indeed, in the composite average of the 37 anomalous warming events (Section 2.6; red triangles in Figure 3), the maximum anomalous warming (Figure 6a) did occur in a geographically similar area to the positive SST anomaly pattern during the January 2016 warm event (Figure 2a). The area affected by anomalously strong warming was a concave south band ∼1,400 km wide reaching offshore to the northwest (Figure 6a). There was a smaller (∼550 km across) and weaker patch of anomalous cooling to the southwest of the band of warming, about 1,300 km offshore. The strongest anomalous warming was concentrated in an area northwest of Punta Lavapié within ∼400 km of the coast (Figure 6a), similar to the location of the strongest *SST*′ (Figure 5). Most of the anomalous warming offshore was contained in a band 1,000–1,500 km wide, which is outlined by the black line in Figure 6a. Rates of anomalous warming in the area closest to the coast near Punta Lavapié were greater than 0.25°C dy^−1^, and in the offshore anomalous warming reached rates between 0.05 and 0.15°C dy^−1^.

The small area of negative *∂SST*′/*∂t* on the southwest side of Figure 6a implies that anomalous cooling is common in that area during warming events off Punta Lavapié, although this was not enough cooling to cause a negative SST anomaly *SST*′ (no blue area in Figure 5).

### 3.2 Composite Mean Net Air–Sea Heat Flux Anomaly

The anomalous warming from the net air‐sea heat flux was small, generally below 0.05°C dy^−1^ (Figure 6b). The total rate of anomalous warming was twice that value or more (Figure 6a). The weak anomalous warming from the $Q_{\mathrm{net}}^{\prime }$ term (Figure 6b) affects a somewhat larger area than the area where anomalous warming is observed. The offshore area of significant mean anomalous warming from the net air‐sea heat flux does have a spatial pattern similar to the region of positive *∂SST*′/*∂t*: warming from the net surface heat flux anomaly term is centered in the black contour of total anomalous warming, extending from the upwelling center toward the northwest (Figure 6b). Within several 100 km of the coast, however, the residual in the anomaly heat budget, *R*′, is much greater than the temperature change from $Q_{\mathrm{net}}^{\prime }$ (Figure 6c). Farther offshore, the residual is still substantial, approximately equal to or somewhat greater than $Q_{\mathrm{net}}^{\prime }/\rho_{w}c_{p}h_{0}$, indicating that even in the area well offshore of the upwelling zone, the air‐sea heat flux anomaly explains at most half of the observed warming. In Figure 6b, the gap between positive values and the coast indicates that the composite mean net surface heat flux anomaly $Q_{\mathrm{net}}^{\prime }$ from ERA5 was not significantly different from zero in a narrow band near the coast. We will not focus on that narrow coastal band in more detail because the accuracy of the reanalyzed fluxes in that area is uncertain, given both the model grid resolution and the low availability of satellite observations very near the coast. Overall, air‐sea heat flux anomalies cannot explain the warm SST anomalies.

### 3.3 Possible Effect of Shallower Mixed‐Layer Depth

**Figure 7:**
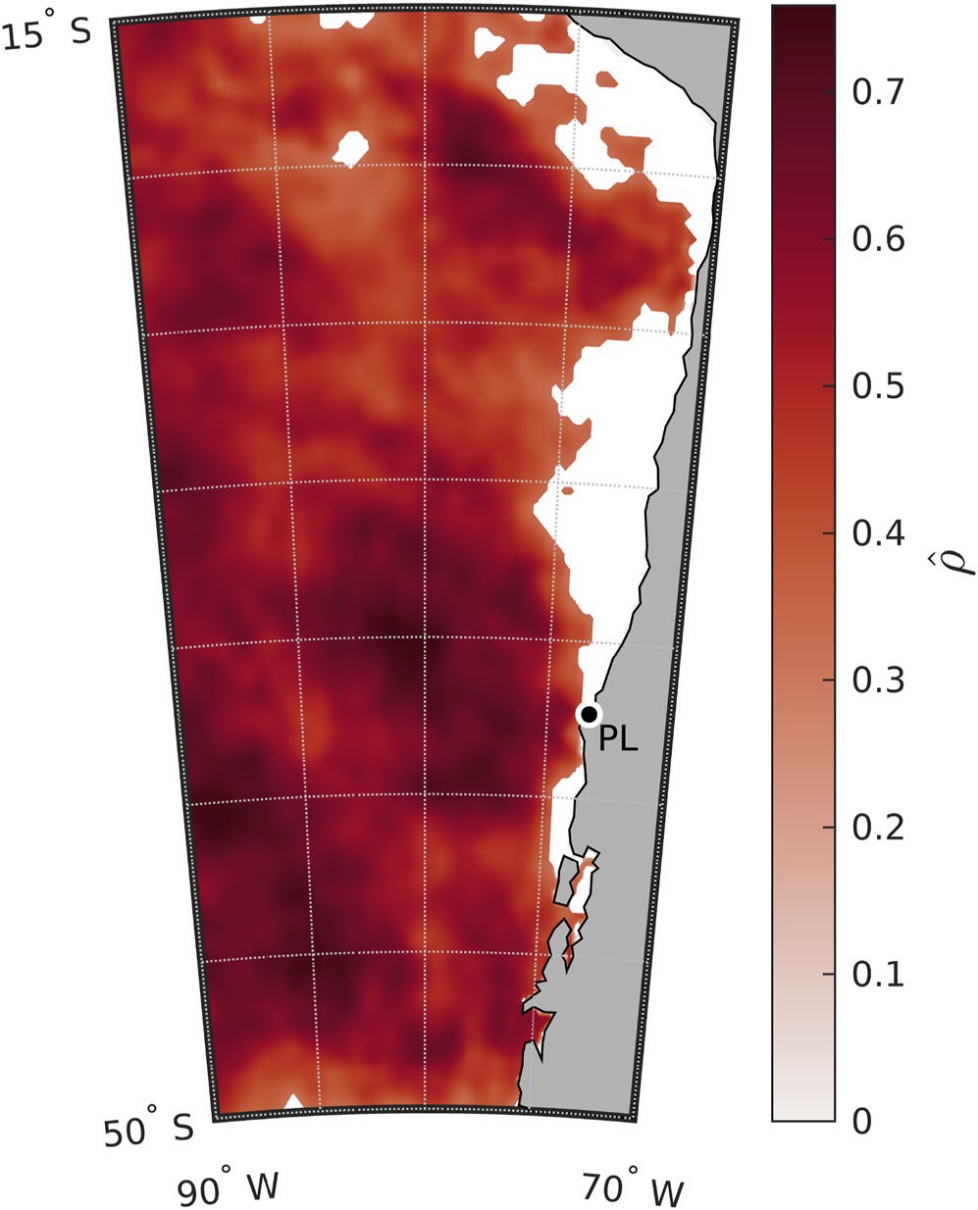
Correlation coefficient $\hat{\rho }$ between the net surface heat flux anomaly $Q_{\mathrm{net}}^{\prime }$ and rate of change of sea surface temperature (SST) anomaly *∂SST*′/*∂t* at the times of peak anomalous warming during the 37 events. White indicates areas where the correlation coefficients are not above the critical value for significance at the 95% confidence level, ${\hat{\rho }}_{\mathrm{crit}}=0.325$. Punta Lavapié is indicated by the black dot enclosed by a white circle and labeled “PL.”.

**Figure 8:**
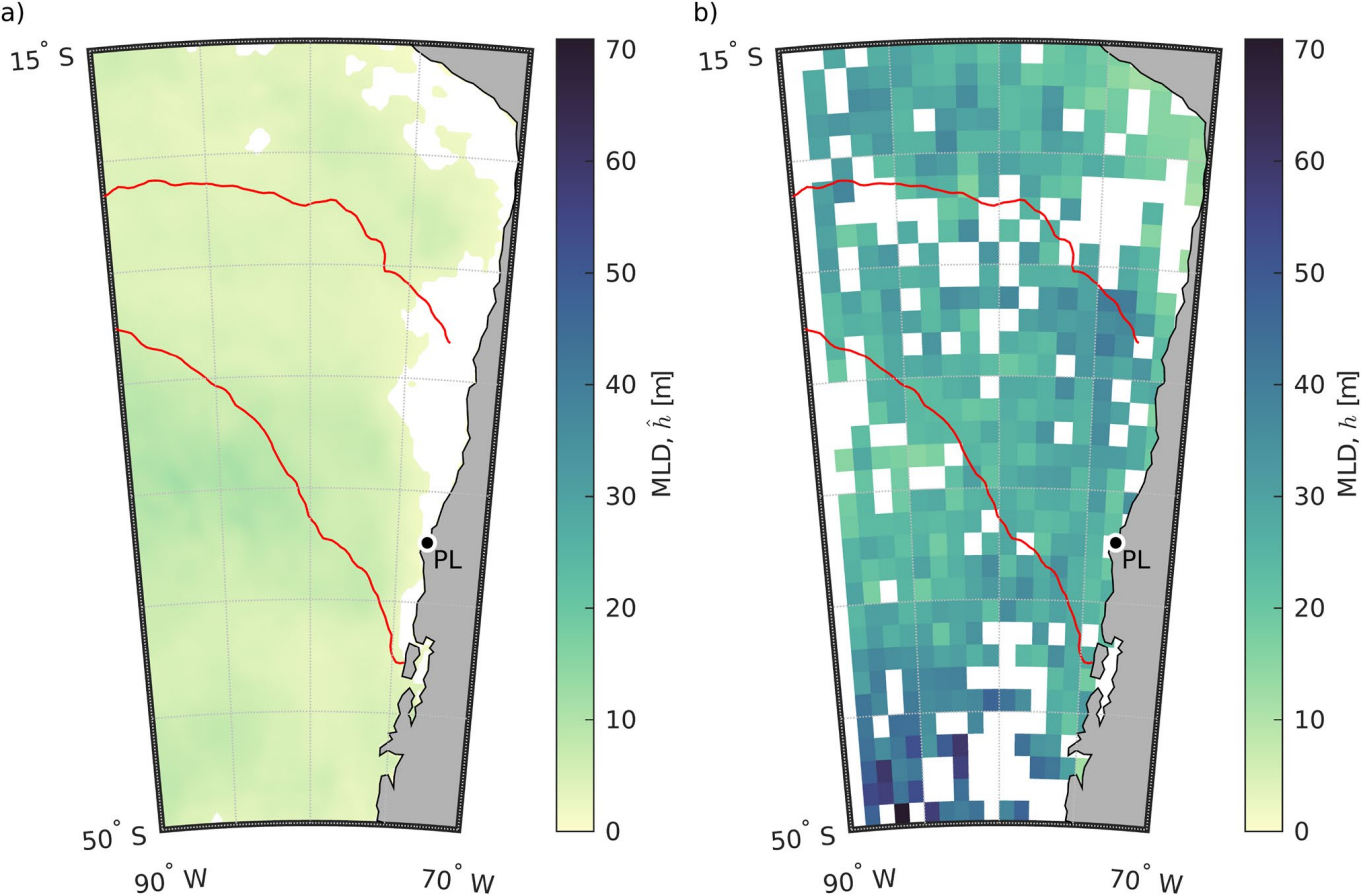
(a) The best‐fit mixed layer depth (MLD) $\hat{h}$ from Equation 7, which is the MLD that would be necessary in the anomaly heat budget (Equation 4) if all anomalous temperature change was due only to the net surface heat flux anomaly absorbed in the mixed layer, that is, if the residual was zero. The white areas are where the skill of the linear regression is less than the critical skill for significance at the 95% confidence level, ${\hat{S}}_{\mathrm{crit}}=0.11$. (b) Seasonal climatology of MLD in summer from Argo float profiles, calculated from Holte et al. (2017) (Section 2.9). The blank squares are where there were not enough Argo profiles within any 1 month to determine a valid MLD climatological value. The red line in each panel shows the outline of the region where *∂SST*′/*∂t* = 0.05°C dy^−1^, the same as the black contour in Figure 6a. In each panel, Punta Lavapié is indicated by the black dot enclosed by a white circle and labeled “PL.”.

Because the magnitude of the surface heat flux term in our anomaly heat budget depends on MLD, we tested whether a shallower MLD is a plausible explanation for the residual. If the MLD was shallower than the climatological MLD *h*_0_ used in Equation 4, then the net surface heat flux anomaly term would explain more of the total anomalous warming than estimated in Figure 6b. To determine how shallow the MLD would need to be in order to explain most or all of the warming, we calculated a best‐fit MLD using a simple model in which the residual in the anomaly heat budget, *R*′, is zero (Section 2.10). The form of this linear regression model was plausible in most of the study area: the correlation between $Q_{\mathrm{net}}^{\prime }$ and *∂SST*′/*∂t* was substantial and greater than the critical value for statistical significance at the 95% confidence level, ${\hat{\rho }}_{\mathrm{crit}}=0.325$ (Figure 7). Only in regions nearest to the coast, where the skill of the model was less than the critical skill ${\hat{S}}_{\mathrm{crit}}=0.11$ (white areas in Figure 8a), were $Q_{\mathrm{net}}^{\prime }$ and *∂SST*′/*∂t* not significantly correlated with 95% confidence. The section of the coast north of Punta Lavapié where the residual was largest in Figure 6c was one such area, so we do not report a best‐fit MLD for the *R*′ = 0 case in that area.

The best‐fit MLDs, that is, the MLDs that would be needed for a shallower mixed layer to explain the residual in the anomaly heat budget, are far shallower than the observed MLDs from Argo float profiles. In the offshore area of anomalous warming (within red contour in Figure 8), the area‐average of the best‐fit MLDs from the linear regressions indicates the MLD would need to be 4.7 ± 0.2 m in order for the composite net surface heat flux anomaly over the 37 warming events to produce the observed temperature change (Figure 8a). This best‐fit MLD is much shallower than the climatological summer MLDs (Figure 8b): the area‐averaged summer climatological MLD within the area of anomalous warming (red contour) is 27.7 ± 0.8 m. The shallowest observed MLDs from individual Argo profiles are also substantially deeper than the best‐fit MLDs from the linear regressions. We examined the individual MLDs provided at http://mixedlayer.ucsd.edu, estimated as in Holte et al. (2017) based on their density algorithm, focusing on the region between 20–40°S and 70–85°W. Among the 4824 profiles available in that region during December–February 2003–2022, there are no Argo MLD estimates shallower than 7 m and only 13 estimates shallower than 8 m. Using the alternative method of a density threshold to define MLD (Holte et al., 2017), there are no MLD estimates shallower than 8 m and only 16 shallower than 9 m. The results from the temperature threshold method are similar (no MLDs < 7 m and only three MLDs < 8 m). Therefore, the best‐fit MLDs, on average ∼5 m as stated above, are extremely shallow (∼5–6 times as shallow) compared to the climatological summer MLDs, and 1.5–2 times as shallow as the shallowest observed MLDs in the record of individual Argo float profiles in the area of anomalous warming.

### 3.4 Wind Stress and Wind Stress Curl Anomalies Preceding Warm Events

**Figure 9:**
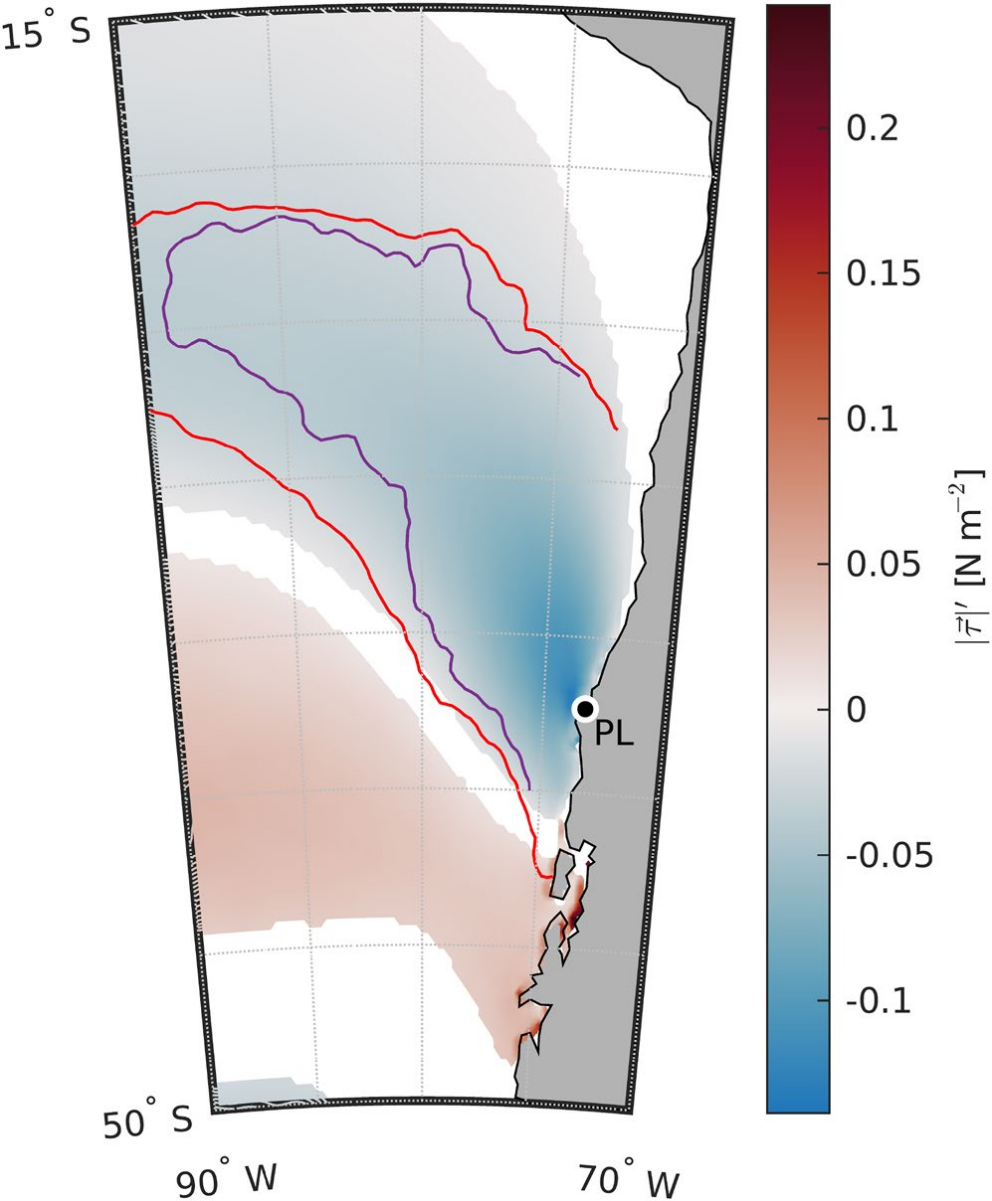
Anomaly in wind stress magnitude associated with the warming events. Color shading: composite average of the (10 days)^−1^ to (6 months)^−1^ band‐pass‐filtered anomaly in wind stress magnitude during the 37 warming events, from the times of peak anomalous warming (red triangles in Figure 3). White areas indicate anomalies not significantly different from zero with 95% confidence. The red line encloses the area where substantial anomalous warming is observed (*∂SST*′/*∂t* ≥ 0.05°C day^−1^, the contour from Figure 6a). The purple line encloses the area where the residual in the anomaly heat budget is substantial (*R*′ ≥ 0.04°C day^−1^). Punta Lavapié is indicated by the black dot enclosed by a white circle and labeled “PL.”.

Because the net air–sea heat flux anomalies did not explain the observed warming events even when we allowed for possible changes in MLD (Sections 3.2 and 3.3), we next examined the role of changes in wind forcing, motivated by studies of analogous warming events in the CCS (Section 1.2). In the area where warming was observed in this study, the composite anomaly in surface wind stress magnitude is negative everywhere, indicating weakened wind stress (blue shading within the red contour in Figure 9). The reduction in wind stress magnitude during the warming events is substantial (0.05–0.1 Pa), especially given that these filtered anomalies have time scales >10 days. Within several hundred km of the coast, the anomaly in wind stress is comparable to the magnitude of the summer climatological mean wind stress (Figure 1), indicating that at the times of peak warming during the development of extreme SST anomalies, the wind stress is close to zero in an area extending hundreds of km to the south, west, and north of Punta Lavapié. South of the area of warming, there is a smaller area of weaker positive anomaly in wind stress magnitude (red shading in Figure 9). The areas of negative and positive wind stress magnitude anomaly are separated by a region of no significant wind stress magnitude anomaly about 40 km wide, indicating that a dipole structure in the wind stress anomaly is associated with these extreme warming events.

**Figure 10:**
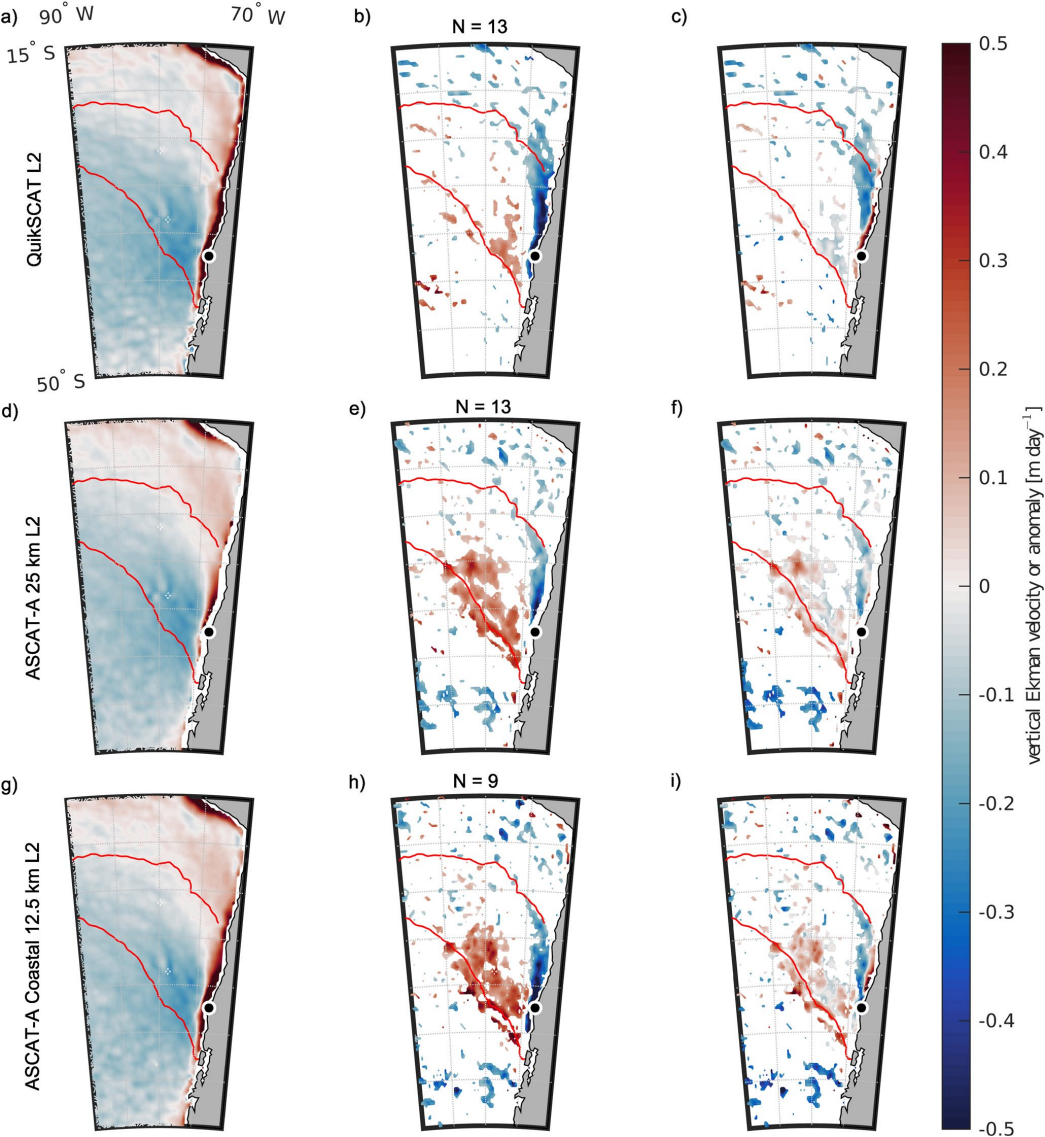
Climatology and anomalies of vertical Ekman pumping velocity based on satellite wind stress curl from QuikSCAT (first row), the ASCAT KNMI 25‐km product (second row), and the ASCAT Coastal Processing 12.5‐km product (third row). The scale for the color shading is the same in all panels. (a, d, and g) The climatological average of vertical Ekman pumping velocity *w*_*Ek*_ over December–February of the years available in each satellite record, that is, the austral summer mean vertical Ekman velocity. Positive *w*_*Ek*_ is defined as upward, contributing to upwelling (Ekman suction), and negative *w*_*Ek*_ is downward, contributing to downwelling (Ekman pumping). Thin white band along the coast: the area where satellite data are not available due to land contamination of the signal. (b, e, and h) Composite average of anomalies in vertical Ekman pumping velocity, $w_{Ek}^{\prime }$, over the warming events (red triangles in Figure 3) captured in the satellite data set in used in that row. Composite anomaly values that are not significantly different from zero with 95% confidence are shown in white. Positive $w_{Ek}^{\prime }$ is defined as upward, indicating more upwelling (Ekman suction), or less downwelling, than in the climatological summer mean, and negative $w_{Ek}^{\prime }$ is downward, indicating less upwelling or more downwelling than in the climatology. (c, f, and i) Sum of the summer mean vertical Ekman pumping velocity from left panels and composite averages over the warming events from middle panels, an estimate of expected *w*_*Ek*_ at the time of peak anomalous warming; sign convention is the same as in the left panels. The number of events contributing to the composites in the middle and right panels of each row is indicated by *N* above the middle panel of that row. As in previous figures, the red contour encloses the area where anomalous warming *∂SST*′/*∂t* ≥ 0.05°C day^−1^ (contour from Figure 6a). In each panel, Punta Lavapié is indicated by the black dot enclosed by a white circle.

Since vertical Ekman pumping or suction can play a role in the mixed‐layer heat budget (Section 2.7), and since anomalies in vertical Ekman velocity were substantial in studies of analogous warming events in the CCS (Section 1.2), we examined the composite anomaly in *w*_*Ek*_ over the 37 warming events. We compared the magnitude and sign of the anomalies to the climatological mean vertical Ekman velocity *w*_*Ek*_ in the same area. The climatological summer vertical Ekman velocity is positive, which would contribute to upwelling, with a magnitude about 0.5 m dy^−1^, in a narrow (∼100–200 km wide) band along the coast north and south of Punta Lavapié (Figures 10a, 10d and 10g, red area above and below black dot). Offshore of that coastal band where the climatological summer wind stress curl contributes to upwelling, the climatological summer Ekman velocity in the area of warming is either negative (Figures 10a, 10d and 10g, blue), which would contribute to downwelling and deepening of the mixed layer, or is weak.

There are two areas of substantial vertical Ekman velocity anomalies during the warming events. The first is a band of strong negative (downward) Ekman velocity anomalies within ∼100–500 km of the coast between 25 and 40°S (Figures 10b, 10e and 10h, blue area north and south of black dot). These downward Ekman velocity anomalies encompass much of the area along the coast where the climatological Ekman velocity is upward and have a similar magnitude to the climatological positive Ekman velocities, but the opposite sign. The wind stress curl anomalies during the warming events therefore tend to cancel the climatological upwelling‐favorable wind stress curl along the coast. The resulting total vertical Ekman velocity during the warming events remains upwelling‐favorable only in a very narrow (∼50–75 km width) band near the coast (Figures 10c and 10i; red area north of black dot). This narrow band is not captured in the wind stress curl computed from the standard KNMI ASCAT‐A 25‐km product (Figure 10f), due to its coarser grid size and wider land mask as compared to the coastal QuikSCAT and KNMI ASCAT‐A Coastal 12.5‐km products (Figures 10c and 10i). Immediately offshore of the narrow band of positive vertical Ekman velocity that persists during the warming events is an area with ∼200–500 km longitudinal extent and ∼1000 km latitudinal extent where the total vertical Ekman velocity becomes negative (downwelling) during the warming events (blue area north of Punta Lavapié in Figures 10c, 10f and 10i).

The second area of substantial vertical Ekman velocity anomalies during the warming events is farther offshore, where the composite Ekman velocity anomalies are positive, the opposite sign from near the coast (Figures 10b, 10e and 10h, red). This indicates either a reduction in wind stress curl‐driven downwelling compared to the climatological value, or a transition to wind stress curl‐driven upwelling, during the warming events. The area of statistically significant positive Ekman velocity anomalies associated with the warming events is much larger in the ASCAT products than the QuikSCAT product (compare red areas in Figures 10e and 10h to red areas in Figure 10b). Because the periods of record of the three satellite wind stress curl products are different (Section 2.1), the differences in area of the positive composite anomalies in Figures 10b, 10e and 10h could be due to either differences in how well each of the satellite products captures wind stress curl anomalies or to differences in the characteristics of warming events that occurred during those periods of record. The composite total Ekman velocities in that offshore area during the warming events indicate a mix of net upward and net downward Ekman velocity, but generally a weak net upward Ekman velocity (Figures 10c, 10f and 10i). The ASCAT products indicate total vertical Ekman velocities during the composite warming event are generally weakly upward (∼0.1 m day^−1^) in a substantial offshore area (red in Figures 10f and 10i) where the climatological vertical Ekman velocity is downward Figures 10d and 10g). This area lies mostly within the area where there is substantial anomalous warming (red contour in Figure 10) and where the residual in the anomaly heat budget is substantial.

Overall, the satellite vector wind stress curl products indicate that during these warming events there is a substantial reduction in wind stress curl‐driven upwelling within 100–200 km of the coast, a transition from curl‐driven upwelling to weak curl‐driven downwelling over a 100s–1,000 km area offshore and to the north of Punta Lavapié, and a transition from curl‐driven downwelling to weak curl‐driven upwelling over an even larger area west and offshore of Punta Lavapié. The strong anomalies in wind stress curl and the equivalent vertical Ekman pumping velocity during the warming events counteract most of the summer climatological pattern, resulting in generally weakened wind stress curl and Ekman pumping velocities, consistent with the wind stress being near zero for 100s–1,000 km around Punta Lavapié during the warming events as discussed above.

## 4 Discussion

### 4.1 Anomalous Net Surface Heat Flux, Residual Warming, and MLD

In the composite warming event, the net surface heat flux anomalies had a spatial structure similar to the observed warming signal *∂SST*′/*∂t* (Figure 6). Nevertheless, the net surface heat flux anomalies could not explain the anomalous warming: the net surface heat flux anomalies (Figure 6b) were insufficient in magnitude to explain the observed warming (Figure 6a). This result depends on the MLD in the mid‐latitude CPCS, which we initially assumed was *h*_0_ = 25 m based on the Holte et al. (2017) climatology. Still, in the area of anomalous warming, the mean summer MLDs are more than 5 times deeper than the MLDs that would be needed to explain the residual 7 (within red outline in Figure 8). Although original Argo profiles did not include many observations in the upper 10 m, the improved vertical sampling resolution available in the MLDs from Holte et al. (2017) could identify MLDs on scales similar to the linearly regressed MLDs if they were present. The mixed layer depth required to explain the residual during the anomalous warming events is ∼2 times shallower than any individual MLD observed in the existing Argo record for this area and season. Therefore, it is not possible for the net surface heat flux anomaly term to explain all of the anomalous warming during our events. Further, although shoaling MLD may play a role in the anomalous warming, mixed‐layer shoaling alone cannot explain the residual in the anomaly heat budget. This suggests that one or more other processes absorbed into the residual of our simplified surface mixed‐layer anomaly heat budget (Equation 4) is a dominant driver in the formation of warm SST anomaly events.

### 4.2 Offshore Warming From Processes Other Than Surface Heat Flux

As mentioned in Section 2.7, the residual, or amount of anomalous warming not explained by the net surface heat flux anomaly, includes *∂SST*′/*∂t* from penetrating shortwave radiation anomalies that are absorbed below the mixed layer, horizontal advection of *SST*′, horizontal eddy diffusion, temporal and advective changes in MLD, and entrainment and mixing with colder water at the base of the mixed layer. Anomalies in penetrating radiation [term (b) in Equation 2] are likely negligible, following the same argument as in Flynn et al. (2017) for the CCS. The shortwave radiative flux anomaly at the surface is already a small part of the net surface heat flux anomaly. Assuming typical absorption coefficients for mid‐latitude coastal or offshore waters (Paulson & Simpson, 1977), shortwave radiation at depth *z* = −*h* is a small fraction, *O*(0.1), of that already small term.

Outside of the upwelling zone, farther than approximately 200–300 km offshore (Bakun & Nelson, 1991; Montecino & Lange, 2009), we do not expect advection by the mean flow or by eddies (terms c and d in Equation 2) to play a large role in the heat budget (Subramanian et al., 2013), so a major contribution to the residual from anomalous advection of MLD or *SST*′ is unlikely. The covariance term (f) is also expected to be negligible in the surface mixed‐layer, where by definition temperature is relatively well‐mixed down to the thermocline.

The effect of processes at the base of the surface mixed‐layer (term e in Equation 2) depends on a MLD that varies spatially and temporally, and the fluid velocity at the base of the mixed layer. Since we do not have sufficient data for the time‐varying MLD, due to Argo floats sampling this area too sparsely and infrequently, and we do not have observations of the velocities at the base of the mixed layer, it is not possible for us to directly estimate the size of anomalies in term e. Term e involves vertical processes at the base of the mixed‐layer: vertical mixing with water below the mixed layer and changes in mixed layer depth, which were inferred to be a substantial contribution to part of the heat budget in the CCS in Flynn et al. (2017). Anomalies in wind stress and wind stress curl can contribute to anomalies in term e: wind stress anomalies can produce anomalies in shear‐driven mixing, entrainment, and mixed layer depth, and anomalies in wind stress curl can produce changes in mixed layer depth (via vertical Ekman velocities). Therefore, our composite averages of the wind stress magnitude anomalies and wind stress curl anomalies at the time of maximum warming provide insight into the potential for anomalies in term e from Equation 2 to explain the residual in the anomaly heat budget (Equation 4).

### 4.3 Wind Stress Anomalies Co‐Located With Anomalous Warming

Entrainment at the base of the mixed‐layer in the mixed‐layer heat budget (term e in Equation 2) is related to the surface wind stress magnitude via shear‐driven vertical mixing (Price et al., 1986). The negative anomalies in wind stress magnitude during warming events (Figure 9) could therefore create anomalies in term e, potentially explaining part of the residual in the anomaly heat budget (Equation 4). Reduced shear‐driven mixing could also lead to shoaling in MLD so that the climatological and anomalous net surface heat fluxes would heat an anomalously shallow mixed layer, resulting in anomalous warming that could explain part of the residual in the heat budget. The section of weak positive wind stress magnitude anomaly over the area of anomalous cooling in the southwest (Figures 6a and 9) is potentially an example of the opposite case in action, with increased wind stress magnitude co‐located with colder SST anomaly.

Nearer to the coast, north of Punta Lavapié, the substantial negative wind stress magnitude anomaly is over some of the area where the net surface heat flux anomaly and the rate of change of *SST*′ were not linearly related (Figure 7) and the linear regression model for best‐fit MLD did not have significant skill (Figure 8). Since in that area near the coast north of Punta Lavapié, changing the MLD could not explain any part of the residual in the anomaly heat budget using only the net surface heat flux anomaly term, there is likely some other process contributing to the warm anomalies in that area that does not scale with the net surface heat flux anomaly, most likely reduced coastal upwelling. The surface wind stress anomaly *SST*′ relationship illustrated by Figure 9 is good motivation for future studies to quantify the contributions of wind stress in the offshore mid‐latitude CPCS surface mixed‐layer anomaly heat budget during anomalously warm events.

### 4.4 Wind Stress Curl Anomalies Co‐Located With Anomalous Warming

Increased (less negative, or positive) vertical Ekman velocities at the base of the mixed‐layer from decreased surface wind stress curl would have a net warming effect on the mixed‐layer temperature in the offshore area where isotherms are not outcropping. The reduction in downward Ekman pumping compared to the climatological conditions would allow mixed‐layer shoaling, and although the air‐sea heat flux anomaly is relatively small (Section 4.1), the positive climatological summer air‐sea heat flux would heat a shallower surface mixed layer (the term representing this effect is incorporated in the residual in Equation 4).

The mean vertical Ekman pumping velocity anomaly $w_{Ek}^{\prime }$ over all of our events has the opposite sign and is on the same order of magnitude of the average summer values in the same region. Especially in the area which is normally in an upwelling regime, $w_{Ek}^{\prime }$ over all events decreases the magnitude of *w*_*Ek*_ toward zero. The area of anomalous warming is over a region with negative $w_{Ek}^{\prime }$ in the north and positive $w_{Ek}^{\prime }$ in the south. The projection of the anomaly on the summer mean shown in Figure 10c, 10f, 10i shows how within this area $w_{Ek}^{\prime }$ would cause typical upwelling regime patterns to tend toward zero and even overall weakly downwelling. The areas where $w_{Ek}^{\prime }$ is significant are also concentrated within ∼100–200 km of the coast, where we expect upwelling to be important. Meanwhile, many anomalies offshore are near zero, implying that changes in the vertical Ekman pumping velocity are less important to the surface mixed layer anomaly heat budget offshore, especially in the northern part of the area of anomalous warming (within the red contour in Figures 10c, 10f and 10i).

During warming events, the higher‐resolution satellite ocean vector wind stress curl fields indicate weakened Ekman suction within ∼100–200 km of the coast. These anomalies suggest that reduced curl‐driven upwelling of cold water may explain part of the large residual within 100–500 km of the coast, consistent with the air‐sea heat flux anomalies being uncorrelated with the observed warming in that area (Figure 7). Offshore of that band, development of downward Ekman pumping in the area 100–400 km offshore of the coast north of Punta Lavapié suggests wind stress curl anomalies contribute to warming by suppressing the normal curl‐driven upwelling (if isotherms are outcropping) or deepening the surface mixed layer and diluting the effect of the climatological summer air‐sea heat flux (if isotherms are not outcropping). Farther offshore, in a 1,000‐km area of anomalous warming, the typical downwelling‐favorable wind stress curl decreases, implying reduced downward Ekman pumping, which would allow mixed‐layer shoaling and amplify the effect of the positive climatological summertime net surface heat flux. Overall, because we expect opposite effects of wind stress curl on *SST*′ depending on whether isotherms are outcropping, both the negative wind stress curl anomalies along the coast and the positive wind stress curl anomalies farther offshore could contribute to anomalous warming.

## 5 Conclusions

Though fisheries in the CPCS are threatened by warm water anomalies, the dominant regional drivers of extreme warm SST anomalies offshore of the major upwelling center at Punta Lavapié on time scales shorter than El Niño events are not well understood. We focused on extreme warm SST anomalies with time scales of 10 days to 6 months. These warm anomalies occurred mostly in austral summer and upwelling season, December through March, and shared a common area of anomalous warming extending ∼2,000 km to the northwest from Punta Lavapié. This anomalous warming could not be fully explained by air‐sea heat flux anomalies, even when allowing for uncertainty in the mixed layer depth. However, the wind stress magnitude was significantly reduced in the area of offshore warming. Further, the vertical Ekman pumping velocities estimated from satellite observations of wind stress curl were significantly reduced or even reversed in sign during the warming events. These observations, combined with approximations of the remaining terms in the offshore ocean surface mixed layer heat budget, imply that reduced entrainment of cold water at the base of the mixed layer and mixed‐layer shoaling are the most plausible drivers of the offshore anomalous warming. The implied mixed‐layer shoaling could be partly due to the change in sign of wind stress curl offshore of the upwelling zone, which indicates a switch from Ekman pumping to Ekman suction during the anomalous warming. In contrast, within ∼100 km of the coast, where isotherms normally outcrop during upwelling, the satellite observations indicate a reduction in the typical Ekman suction, suggesting reduced curl‐driven upwelling could play a role in the anomalous warming. This complex spatial pattern of wind stress curl anomalies, and the substantial size of the anomalies, suggests Ekman pumping anomalies play an underappreciated role in modulating extreme warm water anomalies along and offshore of the CPCS.

The impact of the wind stress and wind stress curl anomalies on SST could be better quantified in future if subsurface data with increased resolution becomes available. Improving the spatial and temporal resolution of observations of ocean surface MLD would help quantify the relative importance of the drivers of anomalous warming that lead to extreme warm SST anomalies. Interesting questions raised by our study that could be addressed as more high spatial and temporal resolution subsurface data become available are (a) is mixed‐layer shoaling consistently observed over the entire area of warming during these warming events? and (b) what is the relative importance of reduced wind stress (entrainment) and reduced wind stress curl (Ekman pumping) in allowing any observed mixed‐layer shoaling?

The tendency of extreme warm events in the CPCS to occur in austral summer (Figure 4) is reminiscent of the anomalous warming events in the CCS that are associated with boreal summer wind relaxations (Section 1.2). The anomalies in wind stress magnitude associated with warming events in the CPCS have a dipole structure (Figure 9), as do the analogous wind relaxation events in the CCS (Section 1.2). The wind stress curl anomalies during the warming events are also qualitatively similar in the CPCS and CCS, with reduced curl‐driven upwelling along the coast and reduced curl‐driven downwelling offshore (Figure 10 and Flynn et al. (2017), their Figures 12 and 13). These similarities in the temporal and spatial patterns of warm SSTs and associated wind stress and wind stress curl anomalies in the two EBUS in the eastern Pacific Ocean, that is, the CPCS and CCS, suggest similar analyses would be fruitful in other EBUS, including the Benguela and Canary/Iberian Current Systems, and could lead to better understanding of anomalously warm events in those systems. Fisheries management in EBUS globally would benefit from improved understanding of the drivers of high SST anomalies other than ENSO, since future anomaly distributions may shift toward current extremes (Field et al., 2012).

## Data Availability

The ERA5 single‐level data used for anomalies of SST, the components of the net surface heat flux, and the wind stress magnitude in the study are available at the ECMWF Copernicus Climate Change Service (C3S) Climate Data Store (CDS) via https://doi.org/10.24381/cds.adbb2d47 with the License to Use Copernicus Products and a free account (Hersbach et al., 2018). The temperature algorithm monthly mean mixed‐layer depth (MLD) data used for the map of summer mean MLD in the study are freely available at mixedlayer.ucsd.edu from the University of California San Diego (Holte et al., 2017, last accessed: 15 June 2021). Design of the PL66 low‐pass filter weights is described in Beardsley et al. (1985), and the code for the PL66 filter is available on GitHub under the MIT License at https://github.com/sea-mat/bobstuff/blob/master/pl66tn.m (Beardsley, 2000). ASCAT‐A L2B scatterometer wind stress data sets used in the wind stress curl calculation (Figures 10d–10i) were obtained from the NASA PO.DAAC via https://podaac.jpl.nasa.gov/dataset/ASCATA-L2-Coastal (Verhoef & Stoffelen, 2013; EUMETSAT/OSI SAF, 2010b) and https://podaac.jpl.nasa.gov/dataset/ASCATA-L2-25km (Verspeek et al., 2010; EUMETSAT/OSI SAF, 2010a). QuikSCAT L2B scatterometer wind stress data were also obtained from the PO.DAAC at https://doi.org/10.5067/QSX12-L2B41 (SeaPAC, 2020).
